# Probiotic-derived postbiotics: a perspective on next-generation therapeutics

**DOI:** 10.3389/fnut.2025.1624539

**Published:** 2025-07-17

**Authors:** Veilumuthu Pattapulavar, Sathiyabama Ramanujam, Bhagyashree Kini, John Godwin Christopher

**Affiliations:** ^1^Department of Biomedical Sciences, School of Biosciences and Technology, Vellore Institute of Technology, Vellore, India; ^2^Faculty of Engineering, Department of Science and Humanities, Karpagam Academy of Higher Education, Coimbatore, India

**Keywords:** gut microbiome, postbiotics, dysbiosis, immunomodulation, next-generation therapeutics

## Abstract

The gut microbiome plays a fundamental role in regulating host immunity, metabolism, and overall health. Disruptions to this microbial ecosystem, known as dysbiosis, have been implicated in various conditions such as colorectal cancer, inflammatory bowel diseases, and metabolic syndromes. Although probiotics are widely used to restore microbial balance, their efficacy is often inconsistent due to variable colonization and concerns over antimicrobial resistance gene transfer. This review explores the growing body of literature surrounding postbiotics—bioactive metabolites produced by probiotics—as a promising alternative to live microbial therapy. We focus on key classes of postbiotics including exopolysaccharides, cell-free supernatants, short-chain fatty acids, and bacteriocins, summarizing their reported immunomodulatory, antimicrobial, antioxidant, and anti-cancer properties. We also highlight recent developments in formulation techniques, such as encapsulation, which enhance their stability and bioavailability. While current findings are promising, limitations persist, including variability in postbiotic composition and a lack of standardized clinical evaluations. Future research should aim to clarify their mechanisms of action, define optimal delivery strategies, and assess long-term safety. Overall, postbiotics present a sustainable, non-viable, and functionally rich alternative to probiotics, aligning with global health goals—Sustainable Development Goal 3 (Good Health and Well-being) and Sustainable Development Goal 9 (Industry, Innovation, and Infrastructure)—by offering safer, scalable, and effective therapeutic solutions.

## 1 Introduction

The gut microbiome is one of the most extensively studied microbiomes, housing a vast number of microorganisms that is 100 times greater than any other bacterial community in the human body, that are pivotal in shaping the host’s immune system and overall health (1, 2). Gut dysbiosis is characterized by disruptions in the composition and function of gut microbial communities—has been associated with a range of conditions, including colorectal cancer (CRC), inflammatory bowel diseases (IBDs), irritable bowel syndrome (IBS), and coeliac disease, among others (2, 3). Alterations in microbial composition—such as reduced abundance of beneficial Firmicutes and Bacteroidetes, and increased levels of pathogenic species like Fusobacterium and Proteobacteria have been observed in IBD and CRC patients. In individuals affected by IBD, a decreased prevalence of anti-inflammatory microbes such as Firmicutes and Bacteroidetes and an increased prevalence of pro-inflammatory taxa like *Fusobacterium* spp. and *Proteobacteria* have been documented by Sokol et al. (4) and Feizi et al. (5). Similar microbial imbalances have been observed in CRC, where specific bacterial drivers such as *Clostridium septicum*, *Streptococcus bovis*, and *Enterococcus faecalis* are implicated in promoting hyperproliferative and genotoxic processes within the gut epithelium (6).

Several factors can lead to gut dysbiosis that include use of antibiotics, stress, poor diet, and infections leading to the loss of beneficial bacteria and dominance of pathogenic bacteria. To treat dysbiosis, a healthy gut microbiome can be restored using fecal microbiota transplantation (FMT), prebiotics, and probiotics (7). Specifically, antibiotic-induced dysbiosis can be alleviated through the introduction of probiotic-rich foods or supplements containing well-characterized strains. Qualified Presumption of Safety (QPS) strains, including *Lactobacillus rhamnosus GG*, *Bifidobacterium longum*, *Lactobacillus plantarum*, and *Saccharomyces boulardii*, have demonstrated effectiveness in restoring gut homeostasis, enhancing mucosal immunity, and reducing inflammation (8). For example, *L. rhamnosus GG* has been shown to prevent antibiotic-associated diarrhea and modulate gut barrier function (9), while *S. boulardii* effectively shortens the duration of acute infectious diarrhea and reduces recurrence rates of *Clostridioides difficile* infections (10). Moreover, probiotic interventions can modulate the gut-brain axis and inflammatory signaling pathways. *Bifidobacterium* and *Lactobacillus* strains have been reported to regulate neurotransmitter metabolism, lower pro-inflammatory cytokines, and improve stress-related symptoms (11).

However, clinical outcomes depend on strain specificity, dosing regimen, duration, and host factors such as baseline microbiota composition and antibiotic exposure (12). Thus, probiotic supplementation with clinically validated QPS strains offers a targeted strategy to mitigate antibiotic-induced dysbiosis, improve gut health, and support systemic immune function under specific conditions where microbial diversity and resilience have been compromised.

This growing recognition of probiotic benefits has encouraged individuals to incorporate probiotic-containing dietary supplements into their routines. These products are valued for their ability to manage gastrointestinal tract infections, inhibit the growth of pathogenic microbes, and alleviate symptoms associated with lactose intolerance.

However, certain probiotic strains commonly found in dietary supplements, including *Enterococcus faecalis*, *Lactobacillus rhamnosus GG*, and *Bifidobacterium animalis subsp. lactis*, have been reported to carry antimicrobial resistance (AMR) genes such as tet(M), erm(B), and vanA. These genes confer resistance to tetracyclines, macrolides, and vancomycin, respectively (13). Horizontal gene transfer, particularly via plasmids and transposons, facilitates the transmission of these resistance genes from probiotics to resident gut microbiota under selective pressure from antibiotic use (14). This mechanism poses a significant risk for the enrichment of multidrug-resistant bacterial populations within the intestinal environment (15). Hence, although probiotics offer health benefits, the potential for AMR gene dissemination warrants careful strain selection and genomic screening prior to probiotic application in clinical and dietary contexts (13).

Despite probiotic use, conclusive evidence of their colonization in the gut mucosa is lacking. The effectiveness of probiotics is highly variable due to individual microbiome differences influenced by factors like diet and medication. Furthermore, stool analysis is not a reliable indicator of probiotic impact on the gut lining. While *in vitro* and mouse studies show some promise, a single human study during colonoscopy failed to demonstrate significant probiotic colonization (12). Recent findings suggest that probiotics’ benefits might come more from the production of metabolites (postbiotics) than from the live bacteria themselves (probiotics) (16).

Recent literature suggests that the beneficial effects attributed to probiotics may arise primarily from their metabolic byproducts, collectively termed postbiotics. Postbiotics, also referred to as cell-free supernatants (CFS), metabolites, or biogenics, are bioactive compounds produced by live probiotic bacteria or released during bacterial lysis. These include diverse substances such as microbial fractions, functional proteins, extracellular polysaccharides, cell wall components (e.g., teichoic acids and peptidoglycans), and short-chain fatty acids (SCFAs) (17, 18). Functionally, postbiotics exert a range of health-promoting effects. They possess immunomodulatory, antimicrobial, antioxidant, and anti-inflammatory properties, which contribute to host defense and intestinal homeostasis. Additionally, certain postbiotic metabolites have been reported to support glucose metabolism, regulate lipid accumulation and obesity, and inhibit pathogenic microorganisms (19). Therefore, postbiotics have the potential to serve as safe next-generation therapeutics without the need to administer live bacteria.

Additionally, postbiotics, including short-chain fatty acids (SCFAs) and bacteriocins, play a vital role in maintaining gut microbial balance and preventing the overgrowth of pathogenic organisms. These metabolites support the integrity of the intestinal barrier and contribute to gut health by promoting the growth of beneficial commensals (17).

Unlike probiotics, postbiotics do not involve the administration of live microorganisms, thereby eliminating the risks associated with AMR gene transfer and the variability of microbial colonization outcomes in different hosts (18). Their inherent stability and targeted effects make them attractive candidates for therapeutic development. This makes them a safer therapeutic option, particularly in immunocompromised or critically ill individuals. Functionally, postbiotics exhibit a diverse range of biological activities, including immunomodulatory, anti-inflammatory, and antimicrobial effects. These properties enable them to be valuable adjuncts in the management of gut dysbiosis, metabolic syndromes, and infectious diseases (20). Furthermore, postbiotics offer substantial industrial potential as stable, non-viable bioactive compounds suitable for incorporation into functional foods, pharmaceuticals, and nutraceuticals. Their production is scalable and sustainable, aligning with global initiatives targeting antibiotic resistance mitigation and supporting innovations in biotechnology and public health (21).

Their stability and efficacy compared to probiotics make them ideal for large-scale industrial applications, fostering advancements in precision medicine, nutraceuticals, and antimicrobial alternatives. Additionally, postbiotic-based products can reduce reliance on antibiotics, aligning with global efforts to combat AMR while promoting sustainable health solutions.

**Figure 1:**
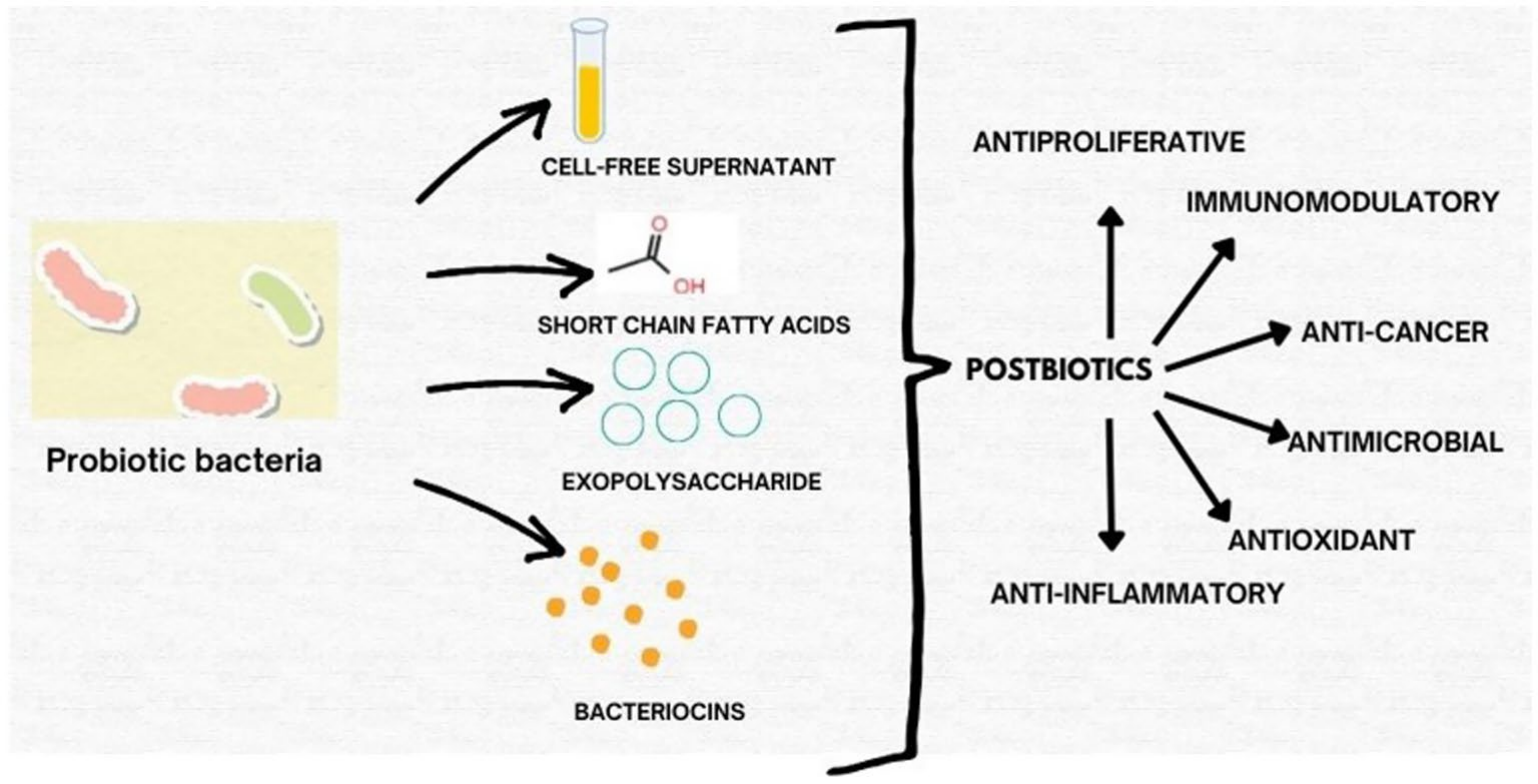
Schematic representation of key classes of postbiotics derived from probiotic bacteria, including exopolysaccharides (EPS), short-chain fatty acids (SCFAs), cell-free supernatants (CFS), and bacteriocins, highlighting their bioactivities.

This perspective aims to consolidate recent findings on the functional roles and mechanisms of postbiotics derived from probiotic bacteria with emphasis on their emerging value as next-generation therapeutics. We discuss their biological activities, clinical relevance, formulation strategies, and potential for industrial translation. By bridging health and innovation, postbiotics support both sustainable development goal 3 (Good Health and Well-Being) and SDG 9 (Industry, Innovation, and Infrastructure), paving the way for safer, more effective, and accessible healthcare solutions (Figure 1).

## 2 Materials and methods

This narrative review was conducted to synthesize current scientific literature on the therapeutic potential of postbiotics derived from probiotic organisms. Relevant articles were identified through comprehensive electronic searches of PubMed, Scopus, ScienceDirect, and Google Scholar databases. The search strategy included combinations of the following keywords: “postbiotics,” “probiotic metabolites,” “cell-free supernatant,” “bacteriocins,” “short-chain fatty acids,” “exopolysaccharides,” “gut microbiome,” “next-generation therapeutics,” and “antimicrobial resistance.”

Over 120 peer-reviewed publications were initially screened. After removing duplicates and applying inclusion criteria, a total of 67 key studies were reviewed in-depth and synthesized to support this article’s discussion. Where appropriate, data from animal models and clinical studies were also included to highlight translational relevance. References were managed using Mendeley and verified against journal citation formats. This perspective follows a narrative approach, emphasizing mechanistic insights, therapeutic relevance, and gaps in current knowledge for future research directions.

## 3 Exopolysaccharides from probiotic bacteria as therapeutic agents

Exopolysaccharides (EPSs) are carbohydrate polymers produced by probiotic bacteria, typically composed of repeating monosaccharide units with molecular weights ranging from 10 to 2000 kDa. These bioactive compounds have demonstrated diverse therapeutic properties, including antioxidant, immunomodulatory, antithrombotic, anticoagulant, anticancer, and wound healing activities (22).

### 3.1 Antioxidant activity

EPSs derived from *Lactobacillus plantarum* RJF4 demonstrated antioxidant potential, achieving 23.63% DPPH radical scavenging activity and 32% total antioxidant capacity (23). Similarly, *Lactobacillus paracasei* EPS isolated from homemade sauerkraut exhibited robust antioxidant effects, with 76.34% total antioxidant capacity, 71.15% reducing power, 68.65% hydrogen peroxide scavenging efficiency, and 60.31% DPPH scavenging activity (24). *Bacillus albus* DM-15, isolated from the Ayurvedic formulation Dasamoolarishta, also displayed DPPH radical scavenging activity across concentrations of 0.5–30 mg/mL (25). EPSs are extracellular carbohydrate polymers secreted by many lactic acid bacteria. Their structure, molecular weight, and monosaccharide composition significantly influence their bioactivity. EPSs derived from *Lactobacillus plantarum* RJF4, for example, demonstrate free radical scavenging capacity and α-amylase inhibition, suggesting potential applications in oxidative stress-related diseases and diabetes (23).

### 3.2 Anticancer properties

Several EPSs exhibit antiproliferative effects against cancer cell lines. *Lactobacillus plantarum* RJF4 EPS demonstrated cytotoxicity toward the MiaPaCa2 pancreatic cancer cell line (26). *Bacillus albus* DM-15 EPS showed notable cytotoxic effects against the lung cancer cell line A549, with an IC50 value of 20 ± 0.97 μg/mL (25). Additionally, *Lactobacillus pantheris* TCP102 produced three EPSs (EPS1–EPS3) that inhibited the proliferation of gastric (BCG-803), colon (HCT-116), and ovarian (A-2780) cancer cells (26). EPSs produced by *Lactobacillus pantheris* TCP102 have been shown to stimulate IL-6 and TNF-α production in macrophages, while also suppressing proliferation of multiple cancer cell lines (26). Their antitumor potential may be attributed to activation of the NF-κB and MAPK signaling pathways, although further mechanistic studies are warranted.

### 3.3 Immunomodulatory activity

EPSs from *Lactobacillus pantheris* TCP102 stimulated pro-inflammatory cytokines such as IL-6 and TNF-α, as well as nitric oxide production in macrophages and Ana-1 cells (26), indicating potential for immune regulation and adjunctive therapy in immunodeficiency disorders.

### 3.4 Wound healing effects

An EPS derived from *Lactiplantibacillus plantarum* EI6 exhibited significant wound-healing capacity. In a scratch assay, EPS-treated cells achieved 50% wound closure, compared to 41% in untreated controls (27). EPSs from probiotic bacteria exhibit multifaceted therapeutic potential and present a viable alternative to the administration of live probiotics for functional food, pharmaceutical, and biomedical applications.

## 4 Cell-free supernatants from probiotic Bacteria as therapeutic agents

**Figure 2:**
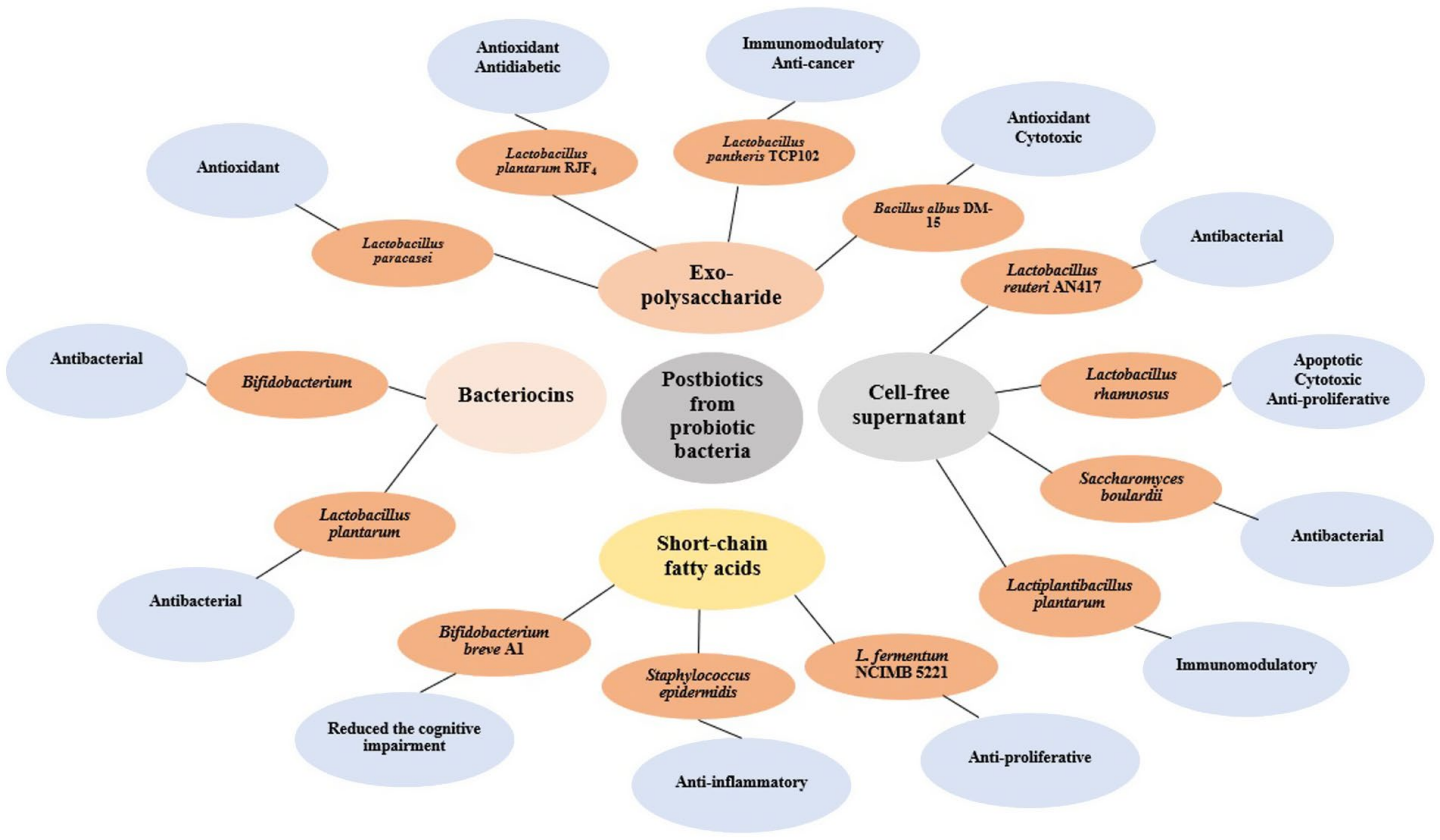
Shematic representation of major classes of postbiotics derived from various probiotic bacteria. The illustration includes exopolysaccharides (EPS) produced by *Lactobacillus plantarum*, *Lactobacillus rhamnosus*, and *Bifidobacterium breve*; bacteriocins produced by *Lactobacillus acidophilus* and *Lactococcus lactis*; cell-free supernatants (CFS) from *Lactobacillus casei*, *Streptococcus thermophilus*, and *Saccharomyces boulardii*; and short-chain fatty acids (SCFAs) such as acetate, propionate, and butyrate commonly released by *Bifidobacterium* and *Faecalibacterium* species. These postbiotics are associated with diverse bioactivities including antioxidant, antibacterial, anti-inflammatory, cytotoxic, and immunomodulatory effects.

Cell-free supernatants (CFS) produced by probiotic bacteria contain secreted bioactive compounds with potent biological activities. These supernatants demonstrate antimicrobial, anticancer, and immunomodulatory properties, making them promising alternatives to conventional probiotics for therapeutic use (28). The Figure 2 provides a schematic representation of postbiotics derived from different probiotic bacteria, including exopolysaccharides, bacteriocins, cell-free supernatants, and short-chain fatty acids. The figure highlights their respective microbial sources such as *Lactobacillus plantarum*, *Bifidobacterium breve*, and *Saccharomyces boulardii*, and their associated bioactivities like antioxidant, anti-inflammatory, antibacterial, cytotoxic, and immunomodulatory effects. This integrative visual framework complements the discussion by summarizing the diverse therapeutic roles of postbiotics.

### 4.1 Anticancer effects

CFS from *Lactobacillus rhamnosus* has shown apoptotic, cytotoxic, and anti-proliferative effects on HT-29 colon cancer cells in a dose- and time-dependent manner. Gene expression analysis revealed increased Bax, caspase-3, and caspase-9 levels, indicating induction of apoptosis and suggesting its potential as a biological anticancer agent (29). Similarly, both live *Lactobacillus casei* and its CFS demonstrated anti-proliferative effects against CT26 and HT29 colon cancer cells (30).

### 4.2 Antimicrobial activity

Several studies have reported the antimicrobial potential of probiotic-derived CFS. The purified CFS from *Lactobacillus rhamnosus* L60 and *Lactobacillus fermentum* L23 produced inhibition zones of 19.01 ± 2.69 mm and 18.33 ± 2.48 mm, respectively, against *Neisseria gonorrhoeae*, with all tested strains displaying complete susceptibility (31). Similarly, the cell-free spent medium (CFSM) from four lactic acid bacteria (LAB) strains—*Lactobacillus acidophilus*, *Lactobacillus delbrueckii*, *Lactobacillus johnsonii*, and *Lactiplantibacillus plantarum*—exhibited strong antimicrobial effects against *Pseudomonas aeruginosa* strains 27853™ and 9027™ (32). In addition, the CFS from *Saccharomyces boulardii* metabolites demonstrated significant antibacterial activity, particularly against *Escherichia coli* (15.43 ± 0.26 mm), with efficacy persisting at pH ≤ 4 and reduced at higher pH values (33). Protease treatment partially diminished activity, indicating the presence of both proteinaceous and non-protein antimicrobial compounds (34).

### 4.3 Immunomodulatory properties

CFS can also modulate immune responses. *Lactiplantibacillus plantarum* CFS was shown to regulate cytokine activity in human macrophage cultures by increasing TNF-α production, suppressing pro-inflammatory IL-8 and TNF-α in response to inflammatory stimuli, and enhancing anti-inflammatory IL-10 levels (34). Additionally, *Lactobacillus reuteri* AN417 CFS demonstrated antimicrobial and anti-biofilm effects against oral pathogens, reducing biofilm integrity, downregulating virulence gene expression, and lowering ATP levels. Minimal inhibitory volumes varied by pathogen: 30% for *Streptococcus mutans*, 20% for *Fusobacterium nucleatum*, and 10% for *Porphyromonas gingivalis* (35). These findings collectively underscore the broad-spectrum biological activities of probiotic-derived CFS, including antimicrobial, anticancer, and immune-regulatory properties. By eliminating the need for live bacterial administration, CFS offers a safer, more controlled, and potentially scalable option for functional food and pharmaceutical applications. Continued research is warranted to isolate, characterize, and optimize these bioactive metabolites for clinical use.

## 5 Short-chain fatty acids from probiotic bacteria as therapeutic agents

Short-chain fatty acids (SCFAs) such as butyrate, propionate, and acetate are primary metabolic end-products generated by the gut microbiome through the fermentation of complex dietary carbohydrates, particularly dietary fibers. This process involves carbohydrate-active enzymes absent in mammals and also produces organic acids and gasses as secondary metabolites (36). SCFAs exert diverse physiological effects, playing essential roles in gut barrier integrity, immunomodulation, and energy metabolism.

### 5.1 Neuroprotective effects

Emerging evidence suggests that SCFAs may reduce the frequency of seizures and alleviate psychiatric comorbidities in individuals with epilepsy. By modulating oxidative stress, balancing neurotransmitter levels, and influencing psychosocial parameters, SCFAs help address depression, anxiety, and seizure susceptibility. Dietary strategies aimed at enhancing SCFA production have been proposed as potential therapeutic interventions for neurological disorders (37). Probiotic-derived SCFAs have emerged as important mediators in the microbiota–gut–brain axis, exerting neuroprotective effects by modulating immune and inflammatory pathways. Recent studies suggest that butyrate and propionate can influence neurological health through G-protein-coupled receptor (GPR109A)-dependent anti-inflammatory mechanisms and by promoting M2 macrophage polarization, which contributes to neuroprotection in neurodegenerative disorders (38). Additionally, SCFA-producing probiotic strains such as *Lactobacillus*, *Bifidobacterium*, and *Clostridium* species have demonstrated potential psychobiotic effects, reducing anxiety, depression, and cognitive impairment via modulation of the microbiota–gut–brain axis (39). A recent comprehensive review in Signal Transduction and Targeted Therapy (40) further emphasizes the role of microbial metabolites, including SCFAs, in regulating neuroinflammation and glial cell function, proposing them as actionable targets for neurodegenerative disease management.

### 5.2 Anticancer properties

Probiotic-derived SCFAs have demonstrated selective anticancer activity, particularly in colorectal cancer (CRC) models. In a comparative study, *Lactobacillus fermentum* NCIMB 5221 exhibited stronger anti-proliferative effects against CRC cells than *Lactobacillus rhamnosus* and *Lactobacillus acidophilus*. This effect was attributed to elevated levels of SCFAs, including butyrate, propionate, and acetate, which induced apoptosis in malignant cells while preserving normal colonocyte viability (41).

### 5.3 Dermatological applications

Within the skin microbiome, the probiotic *Staphylococcus epidermidis* ferments glycerol, generating butyric acid. In murine models, topical application of butyric acid or *S. epidermidis* with glycerol effectively suppressed UVB-induced inflammation by lowering IL-6 expression. Notably, this anti-inflammatory effect was abolished upon blocking the free fatty acid receptor 2 (FFAR2), confirming its role as a mediator of SCFA-driven dermatological benefits (42). SCFAs also contribute to skin health through their role in the gut–skin axis. They have been shown to modulate skin inflammation and may alleviate conditions such as atopic dermatitis, psoriasis, urticaria, and acne. *Staphylococcus epidermidis*, a commensal skin probiotic, produces SCFAs like butyrate and propionate that inhibit *Cutibacterium acnes*-induced inflammation by activating GPR41 and suppressing histone deacetylase (HDAC) activity (43, 44). Moreover, recent findings highlight the broader regulatory roles of gut-derived SCFAs on systemic and cutaneous immunity, offering novel therapeutic avenues for inflammatory skin diseases (45). These insights reinforce the potential of probiotic-derived SCFAs in dermatological and cosmetic formulations targeting inflammatory skin conditions.

### 5.4 Cognitive health benefits

SCFAs also appear to benefit cognitive function. In an Alzheimer’s disease mouse model, acetate derived from *Bifidobacterium breve* strain A1 attenuated cognitive decline. The observed neuroprotective effects were linked to the modulation of inflammatory and neurochemical pathways associated with memory and learning processes (11). In summary, probiotic-derived SCFAs possess broad-spectrum therapeutic potential, contributing to neurological, oncological, dermatological, and cognitive health through diverse bioactive mechanisms.

## 6 Bacteriocins from probiotic bacteria as therapeutic agents

Bacteriocins are ribosomally synthesized antimicrobial peptides produced by probiotic and other bacteria, known for their selective bactericidal or bacteriostatic activity against closely related or pathogenic species (46). Produced primarily by food-grade lactic acid bacteria (LAB), bacteriocins have attracted attention for their potential applications in food preservation, human health, and as alternatives to conventional antibiotics amidst rising antimicrobial resistance concerns (47).

### 6.1 Antimicrobial properties in clinical pathogens

Bacteriocin-producing probiotic strains, particularly those within LAB and *Bifidobacterium* genera, have demonstrated significant antimicrobial activity against clinically relevant pathogens. For example, *Bifidobacterium* species isolated from the human gut inhibited *Staphylococcus aureus* ATCC 25923, *Escherichia coli* B-6954, *Salmonella enterica* ATCC 14028, and *Clostridium tyrobutyricum* LMG, effects attributed to bacteriocin production (48). This pathogen suppression suggests their utility in both gastrointestinal and extraintestinal infections.

### 6.2 Applications in biofilm-associated infections

Notably, *Lactobacillus plantarum* produces plantaricin A, a well-characterized bacteriocin exhibiting potent antimicrobial activity against *Staphylococcus aureus* MTCC 96, particularly strains adhering to extracellular matrix components such as mucin and collagen (49). This activity highlights the potential of plantaricin A in combating biofilm-associated infections, including those occurring on medical implants, where biofilms contribute to treatment resistance and chronicity.

### 6.3 Therapeutic potential summary

Together, SCFAs and bacteriocins derived from probiotics offer promising avenues for next-generation therapeutics. SCFAs contribute to neurological health, inflammation control, and cancer chemoprevention, while bacteriocins provide effective antimicrobial defense against pathogenic bacteria, including biofilm-forming strains. Their diverse mechanisms of action, high safety profile, and demonstrated efficacy position these postbiotic products as valuable candidates for addressing a range of health challenges in clinical, food safety, and biotechnological contexts (Table 1).

**Table 1:** Therapeutic benefits of probiotic-derived postbiotics.

Sl. No	Type of postbiotic	Probiotic producer	Effect	References
1	Exopolysaccharide (EPS)	*Lactobacillus plantarum* RJF_4_	Antioxidant, antidiabetic Toxicity toward pancreatic cell-line MiaPaCa2	Dilna et al. (23)
2	EPS	*Lactobacillus pantheris* TCP102	Immunomodulatory, anti-cancer	Sheng et al. (26)
3	EPS	*Lactiplantibacillus plantarum* EI6	Wound-healing	Zaghloul et al. (27)
4	EPS	*Bacillus albus* DM-15	Antioxidant, cytotoxicity against the lung cancer line A54	Vinothkanna et al. (25)
5	EPS	*Lactobacillus paracasei*	Antioxidant	Shankar et al. (24)
6	CFS	*Lactobacillus rhamnosus*	Apoptotic, cytotoxic Anti-proliferative against HT-29 colon cancer cell-line	Dehghani et al. (29)
7	CFS	*Lactobacillus casei*	Anti-proliferative effects against CT26 and HT29 colon cancer cells	Tiptiri-Kourpeti et al. (30)
8	CFS	*Lactobacillus rhamnosus* L60 and *L. fermentum* L23	Antibacterial against *Neisseria gonorrheae*	Ruíz et al. (31)
9	CFS	*Lactobacillus acidophilus*, *Lactobacillus delbrueckii*, *Lactobacillus johnsonii*, and *Lactiplantibacillus plantarum*	Antibacterial against *Pseudomonas aeruginosa* strains 27853™ and 9027™	Drumond et al. (32)
10	CFS	*Lactiplantibacillus plantarum*	Immunomodulatory	Rocchetti et al. (34)
11	CFS	*Lactobacillus reuteri* AN417	Antibacterial against oral pathogens *Streptococcus mutans*, *Fusobacterium nucleatum*, *Porphyromonas gingivalis*	Yang et al. (35)
12	CFS	*Saccharomyces boulardii*	Antibacterial against *Escherichia coli*	Fu et al. (33)
13	SCFA	*Lactobacillus fermentum* NCIMB 5221	Anti-proliferative effect on colorectal cancer cells	Meenakshi et al. (41)
14	SCFA	*Staphylococcus epidermidis*	Anti-inflammatory	Keshari et al. (42)
15	SCFA	*Bifidobacterium breve* A1	Reduced the cognitive impairment in mice with Alzheimer’s Disease	Kobayashi et al. (11)
16	Bacteriocin	*Bifidobacterium*	Antibacterial against *Staphylococcus aureus* ATCC 25923, *Escherichia coli* B-6954, *Salmonella enterica* ATCC 14028, and *Clostridium tyrobutyricum* LMG	Prosekov et al. (48)
17	Bacteriocin (Plantaricin A)	*Lactobacillus plantarum*	Antimicrobial activity against *Staphylococcus aureus* MTCC	Mukherjee and Ramesh (49)
18	EPS	*Leuconostoc mesenteroides LM187*	Antioxidant activity	Zhang et al. (50)
19	SCF	*Bifidobacterium adolescentis*	Antidiabetic	Qian et al. (51)
20	CFS	*Lactiplantibacillus plantarum T1*	Anti-inflammatory	Hao et al. (52)
21	Bacteriocin	*Enterococcus faecium*	Anti listgerial activity	Meral-Aktaş et al. (53)

EPS, Exopolysaccharide; CFS, Cell-Free Supernatant; SCFA, Short-Chain Fatty Acid; HT-29, Human Colorectal Adenocarcinoma Cell Line; CT26, Mouse Colon Carcinoma Cell Line; MiaPaCa2, Human Pancreatic Cancer Cell Line; A549, Human Lung Carcinoma Cell Line; *S. aureus*, *Staphylococcus aureus*; E*. coli*, *Escherichia coli*; *P. aeruginosa*, *Pseudomonas aeruginosa*; S*. mutans*, *Streptococcus mutans*; *F. nucleatum*, *Fusobacterium nucleatum*; *P. gingivalis*, *Porphyromonas gingivalis*.

## 7 Conclusion

Postbiotics derived from probiotics represent an emerging frontier in next-generation therapeutics. By harnessing non-viable microbial components such as exopolysaccharides, cell-free supernatants, short-chain fatty acids, and bacteriocins, they have demonstrated diverse bioactivities including antimicrobial, anti-inflammatory, immunomodulatory, and anticancer effects. Due to their stability and targeted action, postbiotics may help overcome key limitations associated with live probiotics, such as inconsistent colonization and the risk of antimicrobial resistance gene transfer. Unlike probiotics that rely on active colonization, postbiotics act through their metabolites, offering more predictable outcomes. However, their transient effects may require enhancement through formulation technologies. Approaches such as nanoemulsions, hydrogels, liposomes, and polymer-based delivery systems are being explored to improve their stability, controlled release, and bioavailability in the gut. As research progresses, postbiotics are being positioned as a reliable adjunct or alternative to traditional probiotic therapy. Their development aligns with broader global health goals—specifically SDG 3 (Good Health and Well-being) by contributing to alternatives to antibiotics, and SDG 9 (Industry, Innovation, and Infrastructure) through their role in advancing biotechnological innovation. Overall, postbiotics hold promising potential for application in functional foods, pharmaceuticals, and precision medicine, supporting a sustainable and accessible future in healthcare.

## Data Availability

The original contributions presented in the study are included in the article/supplementary material, further inquiries can be directed to the corresponding author.

## References

[1] Dahiya D Nigam PS. Antibiotic-therapy-induced gut dysbiosis affecting gut microbiota—brain axis and cognition: restoration by intake of probiotics and synbiotics. Int J Mol Sci. (2023) 24:3074. doi: 10.3390/ijms24043074, PMID: 36834485 PMC9959899

[2] Winter SE Bäumler AJ. Gut dysbiosis: ecological causes and causative effects on human disease. Proc Natl Acad Sci USA. (2023) 120:e2316579120. doi: 10.1073/pnas.2316579120, PMID: 38048456 PMC10722970

[3] De Vos WM Tilg H Van Hul M Cani PD. Gut microbiome and health: mechanistic insights. Gut. (2022) 71:1020–32. doi: 10.1136/gutjnl-2021-326789, PMID: 35105664 PMC8995832

[4] Sokol H Leducq V Aschard H Pham HP Jegou S Landman C. Fungal microbiota dysbiosis in IBD. Gut. (2017) 66:1039–48. doi: 10.1136/GUTJNL-2015-310746, PMID: 26843508 PMC5532459

[5] Feizi H Rezaee MA Ghotaslou R Sadrkabir M Jadidi-Niaragh F Gholizadeh P. Gut microbiota and colorectal Cancer risk factors. Curr Pharm Biotechnol. (2022) 24:1018–34. doi: 10.2174/1389201023666221005103340, PMID: 36200153

[6] Lau AWY Tan LTH Ab Mutalib NS Wong SH Letchumanan V Lee LH. The chemistry of gut microbiome in health and diseases. Prog Microb Mol Biol. (2021) 4:a0000175. doi: 10.36877/pmmb.a0000175

[7] Acevedo-Román A Pagán-Zayas N Velázquez-Rivera LI Torres-Ventura AC Godoy-Vitorino F. Insights into gut dysbiosis: inflammatory diseases, obesity, and restoration approaches. Int J Mol Sci. (2024) 25:9715. doi: 10.3390/ijms25179715, PMID: 39273662 PMC11396321

[8] Hill C Guarner F Reid G Gibson GR Merenstein DJ Pot B. Expert consensus document: the international scientific association for probiotics and prebiotics consensus statement on the scope and appropriate use of the term probiotic. Nat Rev Gastroenterol Hepatol. (2014) 11:506–14. doi: 10.1038/NRGASTRO.2014.66, PMID: 24912386

[9] Szajewska H Canani RB Guarino A Hojsak I Indrio F Kolacek S. Probiotics for the prevention of antibiotic-associated diarrhea in children. J Pediatr Gastroenterol Nutr. (2016) 62:495–506. doi: 10.1097/MPG.0000000000001081, PMID: 26756877

[10] McFarland LV. Systematic review and meta-analysis of *Saccharomyces boulardii* in adult patients. World J Gastroenterol. (2010) 16:2202–22. doi: 10.3748/WJG.V16.I18.2202, PMID: 20458757 PMC2868213

[11] Kobayashi Y Sugahara H Shimada K Mitsuyama E Kuhara T Yasuoka A. Therapeutic potential of *Bifidobacterium breve* strain A1 for preventing cognitive impairment in Alzheimer’s disease. Sci Rep. (2017) 7:1–10. doi: 10.1038/s41598-017-13368-2 29044140 PMC5647431

[12] Zmora N Zilberman-Schapira G Suez J Mor U Dori-Bachash M Bashiardes S. Personalized gut mucosal colonization resistance to empiric probiotics is associated with unique host and microbiome features. Cell. (2018) 174:1388–1405.e21. doi: 10.1016/J.CELL.2018.08.041, PMID: 30193112

[13] Zheng M Zhang R Tian X Zhou X Pan X Wong A. Assessing the risk of probiotic dietary supplements in the context of antibiotic resistance. Front Microbiol. (2017) 8:264345. doi: 10.3389/FMICB.2017.00908 PMC5437161 28579981

[14] Huddleston JR. Horizontal gene transfer in the human gastrointestinal tract: potential spread of antibiotic resistance genes. Infect Drug Resist. (2014) 7:167–76. doi: 10.2147/IDR.S48820, PMID: 25018641 PMC4073975

[15] Tan R Jin M Li J Yang D. The dissemination, health risks, and mitigation approaches of antibiotic resistance genes in the gut microbiome. J Hazard Mater Adv. (2025) 17:100634. doi: 10.1016/J.HAZADV.2025.100634

[16] Prajapati N Patel J Singh S Yadav VK Joshi C Patani A. Postbiotic production: harnessing the power of microbial metabolites for health applications. Front Microbiol. (2023) 14:1306192. doi: 10.3389/fmicb.2023.1306192, PMID: 38169918 PMC10758465

[17] Aguilar-Toalá JE Garcia-Varela R Garcia HS Mata-Haro V González-Córdova AF Vallejo-Cordoba B. Postbiotics: an evolving term within the functional foods field. Trends Food Sci Technol. (2018) 75:105–14. doi: 10.1016/J.TIFS.2018.03.009

[18] Tsilingiri K Rescigno M. Postbiotics: what else? Benef Microbes. (2013) 4:101–7. doi: 10.3920/BM2012.0046 23271068

[19] Liang B Xing D. The current and future perspectives of postbiotics. Probiot Antimicrob Proteins. (2023) 15:1626–43. doi: 10.1007/s12602-023-10045-x, PMID: 36763279 PMC9913028

[20] Wegh CAM Geerlings SY Knol J Roeselers G Belzer C. Postbiotics and their potential applications in early life nutrition and beyond. Int J Mol Sci. (2019) 20:4673. doi: 10.3390/IJMS20194673, PMID: 31547172 PMC6801921

[21] Taverniti V Guglielmetti S. The immunomodulatory properties of probiotic microorganisms beyond their viability (ghost probiotics: proposal of paraprobiotic concept). Genes Nutr. (2011) 6:261–74. doi: 10.1007/S12263-011-0218-X, PMID: 21499799 PMC3145061

[22] Birch J Van Calsteren MR Pérez S Svensson B. The exopolysaccharide properties and structures database: EPS-DB. Application to bacterial exopolysaccharides. Carbohydr Polym. (2019) 205:565–70. doi: 10.1016/j.carbpol.2018.10.063, PMID: 30446142

[23] Dilna SV Surya H Aswathy RG Varsha KK Sakthikumar DN Pandey A. Characterization of an exopolysaccharide with potential health-benefit properties from a probiotic *Lactobacillus plantarum* RJF4. LWT. (2015) 64:1179–86. doi: 10.1016/J.LWT.2015.07.040

[24] Shankar T Palpperumal S Kathiresan D Sankaralingam S Balachandran C Baskar K. Biomedical and therapeutic potential of exopolysaccharides by *Lactobacillus paracasei* isolated from sauerkraut: screening and characterization. Saudi J Biol Sci. (2021) 28:2943–50. doi: 10.1016/j.sjbs.2021.02.030 34025171 PMC8117039

[25] Vinothkanna A Sathiyanarayanan G Rai AK Mathivanan K Saravanan K Sudharsan K. Exopolysaccharide produced by probiotic Bacillus albus DM-15 isolated from Ayurvedic fermented Dasamoolarishta: characterization, antioxidant, and anticancer activities. Front Microbiol. (2022) 13:832109. doi: 10.3389/FMICB.2022.832109, PMID: 35308379 PMC8927020

[26] Sheng S Fu Y Pan N Zhang H Xiu L Liang Y. Novel exopolysaccharide derived from probiotic *Lactobacillus pantheris* TCP102 strain with immune-enhancing and anticancer activities. Front Microbiol. (2022) 13:1015270. doi: 10.3389/FMICB.2022.1015270, PMID: 36225355 PMC9549278

[27] Zaghloul EH Ibrahim MIA. Production and characterization of exopolysaccharide from newly isolated marine probiotic *Lactiplantibacillus plantarum* EI6 with *in vitro* wound healing activity. Front Microbiol. (2022) 13:903363. doi: 10.3389/FMICB.2022.903363, PMID: 35668753 PMC9164304

[28] Pelyuntha W Chaiyasut C Kantachote D Sirilun S. Cell-free supernatants from cultures of lactic acid bacteria isolated from fermented grape as biocontrol against salmonella Typhi and *Salmonella Typhi*murium virulence via autoinducer-2 and biofilm interference. PeerJ. (2019) 7:e7555. doi: 10.7717/PEERJ.7555, PMID: 31523511 PMC6715067

[29] Dehghani N Tafvizi F Jafari P. Cell cycle arrest and anti-cancer potential of probiotic *Lactobacillus rhamnosus* against HT-29 cancer cells. Bioimpacts. (2021) 11:245–52. doi: 10.34172/BI.2021.32, PMID: 34631486 PMC8494254

[30] Tiptiri-Kourpeti A Spyridopoulou K Santarmaki V Aindelis G Tompoulidou E Lamprianidou EE. *Lactobacillus casei* exerts anti-proliferative effects accompanied by apoptotic cell death and up-regulation of TRAIL in colon carcinoma cells. PLoS One. (2016) 11:e0147960. doi: 10.1371/JOURNAL.PONE.0147960, PMID: 26849051 PMC4744000

[31] Ruíz FO Pascual L Giordano W Barberis L. Bacteriocins and other bioactive substances of probiotic lactobacilli as biological weapons against *Neisseria gonorrhoeae*. Pathog Dis. (2015) 73:ftv013. doi: 10.1093/FEMSPD/FTV013, PMID: 25673666

[32] Drumond MM Tapia-Costa AP Neumann E Nunes ÁC Barbosa JW Kassuha DE. Cell-free supernatant of probiotic bacteria exerted antibiofilm and antibacterial activities against *Pseudomonas aeruginosa*: a novel biotic therapy. Front Pharmacol. (2023) 14:1152588. doi: 10.3389/FPHAR.2023.1152588, PMID: 37397469 PMC10311102

[33] Fu JJ Liu J Wen XP Zhang G Cai J Qiao Z. Unique probiotic properties and bioactive metabolites of *Saccharomyces boulardii*. Probiot Antimicrob Proteins. (2023) 15:967–82. doi: 10.1007/S12602-022-09953-1, PMID: 35608794

[34] Rocchetti MT Russo P De Simone N Capozzi V Spano G Fiocco D. Immunomodulatory activity on human macrophages by cell-free supernatants to explore the probiotic and Postbiotic potential of *Lactiplantibacillus plantarum* strains of plant origin. Probiot Antimicrob Proteins. (2024) 16:911–26. doi: 10.1007/S12602-023-10084-4, PMID: 37202651 PMC11126452

[35] Yang KM Kim JS Kim HS Kim YY Oh JK Jung HW. *Lactobacillus reuteri* AN417 cell-free culture supernatant as a novel antibacterial agent targeting oral pathogenic bacteria. Sci Rep. (2021) 11:1–16. doi: 10.1038/S41598-020-80921-X 33452304 PMC7810884

[36] Martin-Gallausiaux C Marinelli L Blottière HM Larraufie P Lapaque N. Conference on diet and digestive disease symposium 2: sensing and signalling of the gut environment: SCFA: mechanisms and functional importance in the gut. Proc Nutr Soc. (2021) 80:37–49. doi: 10.1017/S0029665120006916 32238208

[37] Kim S Park S Choi TG Kim SS. Role of short chain fatty acids in epilepsy and potential benefits of probiotics and prebiotics: targeting “health” of epileptic patients. Nutrients. (2022) 14:2982. doi: 10.3390/NU14142982, PMID: 35889939 PMC9322917

[38] Kalyanaraman B Cheng G Hardy M. Gut microbiome, short-chain fatty acids, alpha-synuclein, neuroinflammation, and ROS/RNS: relevance to Parkinson’s disease and therapeutic implications. Redox Biol. (2024) 71:103092. doi: 10.1016/J.REDOX.2024.103092, PMID: 38377788 PMC10891329

[39] Cheng Y Liu J Ling Z. Short-chain fatty acids-producing probiotics: a novel source of psychobiotics. Crit Rev Food Sci Nutr. (2022) 62:7929–59. doi: 10.1080/10408398.2021.1920884, PMID: 33955288

[40] Loh JS Mak WQ Tan LKS Ng CX Chan HH Yeow SH. Microbiota–gut–brain axis and its therapeutic applications in neurodegenerative diseases. Signal Transduct Target Ther. (2024) 9:1–53. doi: 10.1038/s41392-024-01743-1 38360862 PMC10869798

[41] Kahouli I Malhotra M Alaoui-Jamali M Prakash S. *In-vitro* characterization of the anti-cancer activity of the probiotic bacterium *Lactobacillus fermentum* NCIMB 5221 and potential against colorectal cancer. J Cancer Sci Ther. (2015) 7:7. doi: 10.4172/1948-5956.1000354

[42] Keshari S Balasubramaniam A Myagmardoloonjin B Herr DR Negari IP Huang CM. Butyric acid from probiotic *staphylococcus epidermidis* in the skin microbiome down-regulates the ultraviolet-induced pro-inflammatory IL-6 cytokine via short-chain fatty acid receptor. Int J Mol Sci. (2019) 20:4477. doi: 10.3390/IJMS20184477, PMID: 31514281 PMC6769796

[43] Xiao X Hu X Yao J Cao W Zou Z Wang L. The role of short-chain fatty acids in inflammatory skin diseases. Front Microbiol. (2023) 13:1083432. doi: 10.3389/FMICB.2022.1083432, PMID: 36817115 PMC9932284

[44] Tegegne BA Kebede B. Probiotics, their prophylactic and therapeutic applications in human health development: a review of the literature. Heliyon. (2022) 8:e09725. doi: 10.1016/J.HELIYON.2022.E09725, PMID: 35785237 PMC9240980

[45] Gao T Wang X Li Y Ren F. The role of probiotics in skin health and related gut–skin axis: a review. Nutrients. (2023) 15:3123. doi: 10.3390/NU15143123, PMID: 37513540 PMC10385652

[46] Ozma MA Abbasi A Akrami S Lahouty M Shahbazi N Ganbarov K. Postbiotics as the key mediators of the gut microbiota-host interactions. Inf Med. (2022) 30:180–93. doi: 10.53854/LIIM-3002-3, PMID: 35693065 PMC9177191

[47] Sugrue I Ross RP Hill C. Bacteriocin diversity, function, discovery and application as antimicrobials. Nat Rev Microbiol. (2024) 22:556–71. doi: 10.1038/S41579-024-01045-X, PMID: 38730101 PMC7616364

[48] Prosekov AY Dyshlyuk LS Milentyeva IS Sykhikh SA Babich OO Ivanova SA. Antioxidant and antimicrobial activity of bacteriocin-producing strains of lactic acid bacteria isolated from the human gastrointestinal tract. Prog Nutr. (2017) 19:67–80. doi: 10.23751/PN.V19I1.5147

[49] Mukherjee S Ramesh A. Bacteriocin-producing strains of *Lactobacillus plantarum* inhibit adhesion of *Staphylococcus aureus* to extracellular matrix: quantitative insight and implications in antibacterial therapy. J Med Microbiol. (2015) 64:1514–26. doi: 10.1099/JMM.0.000181, PMID: 26445850

[50] Zhang Q Wang J Sun Q Zhang SM Sun XY Li CY. Characterization and antioxidant activity of released exopolysaccharide from potential probiotic *Leuconostoc mesenteroides* LM187. J Microbiol Biotechnol. (2021) 31:1144–53. doi: 10.4014/JMB.2103.03055, PMID: 34226411 PMC9705892

[51] Qian X Si Q Lin G Zhu M Lu J Zhang H. *Bifidobacterium adolescentis* is effective in relieving type 2 diabetes and may be related to its dominant core genome and gut microbiota modulation capacity. Nutrients. (2022) 14:2479. doi: 10.3390/NU14122479/S1 35745208 PMC9227778

[52] Hao R Liu Q Wang L Jian W Cheng Y Zhang Q. Anti-inflammatory effect of *Lactiplantibacillus plantarum* T1 cell-free supernatants through suppression of oxidative stress and NF-κB- and MAPK-signaling pathways. Appl Environ Microbiol. (2023) 89:e00608-23. doi: 10.1128/AEM.00608-23 37702501 PMC10617582

[53] Meral-Aktaş H. Bacteriocin characterization of *Enterococcus faecium* isolates and evaluation of their in situ anti-Listerial activity in Beyaz cheese. Food Biosci. (2024) 61:104741. doi: 10.1016/J.FBIO.2024.104741, PMID: 40623446

